# Molecular biophysics of viral proteins: From mechanistic insights to microbiological and clinical applications

**DOI:** 10.3934/microbiol.2026030

**Published:** 2026-09-15

**Authors:** Alessandro Paolo Addamo, Floriana D'Angeli, Carlo Genovese

**Affiliations:** Department of Medicine and Surgery, School of Medicine and Surgery, University of Enna “Kore”, Enna, 94100 Italy

**Keywords:** molecular biophysics, viral proteins, cryo-electron microscopy, nuclear magnetic resonance, atomic force microscopy, all-atom molecular dynamics, coarse-grained molecular dynamics, integrative structural biology, virus-like particles, viral tropism

## Abstract

Viral proteins are dynamic molecular machines that couple molecular-scale events to microbiological phenotypes, including host-cell tropism, entry efficiency, environmental persistence, strain-specific immune escape, and antiviral resistance. In this review, we critically evaluated how experimental, computational, and biochemical approaches define these links and support clinical translation. We organized the evidence around four key observables: Structure, dynamics, mechanics, and thermodynamics. We then assessed which conclusions remained robust across methods and viral families. Nuclear magnetic resonance spectroscopy, cryogenic electron microscopy, atomic force microscopy, calorimetry, all-atom molecular dynamics (MD), coarse-grained MD, structural bioinformatics, artificial intelligence, and biochemical reconstitution were compared in terms of resolution, sampling, assumptions, and validation requirements. Human immunodeficiency virus type 1 and severe acute respiratory syndrome coronavirus 2 were used as data-rich exemplars, but the resulting conclusions were tested against influenza virus, hepatitis B virus (HBV), human papillomavirus (HPV), Ebola virus, adenovirus, bacteriophages, and other systems. Particular attention was given to force-field selection, enhanced sampling, free-energy calculations, convergence, experimentally restrained modeling, and the multiscale relationship between all-atom and coarse-grained simulations. We also examined integrative workflows, the licensed HBV and HPV virus-like particle (VLP) vaccines, emerging dengue and coronavirus VLP platforms, and clinically validated structure-guided antivirals and prefusion-stabilized antigens. The analysis identified where evidence converges, where method-specific biases or limited taxonomic sampling constrain generalization, and which measurements are required to connect a molecular mechanism to infection, transmission-relevant traits, surveillance, or therapeutic performance. This critical, experimentally anchored framework defines the distinctive contribution of the review.

## 1. Introduction

Viral proteins operate under unusually tight constraints: A limited proteome must support entry, replication, assembly, release, and immune evasion while remaining adaptable to mutation [1]–[3]. Molecular biophysics addresses how folding, conformational exchange, allostery, mechanics, and molecular recognition produce these functions [4]–[8]. In this review, a microbiological application is defined as a molecular explanation of a phenotype relevant to viral biology at the host or population level, including host range and cell tropism, entry efficiency, environmental persistence, strain-level variation, immune escape, or the emergence of antiviral resistance. This definition distinguishes microbiological inference from a purely structural description.

Experimental and computational methods observe different projections of the same energy landscape. Cryogenic electron microscopy (cryo-EM) resolves large assemblies and recurrent conformations; nuclear magnetic resonance (NMR) and single-molecule methods quantify exchange and low-population states; atomic force microscopy (AFM) measures mechanical response; and calorimetry reports binding and unfolding energetics [9]–[17]. All-atom and coarse-grained molecular dynamics (MD) complement these measurements by exploring molecular behavior across broader spatial and temporal scales, while structural bioinformatics and machine learning (ML) prioritize hypotheses [18]–[23]. Their outputs are not interchangeable. Each method has characteristic sample requirements, resolution limits, priors, and sources of bias, and mechanistic confidence increases when independent observables converge.

Historical examples show what such convergence can achieve. The foundational analysis of icosahedral symmetry and quasi-equivalence established a physical language for viral capsids [24]. The human immunodeficiency virus type 1 (HIV-1) protease structure [25], together with rational inhibitor design [26], linked an atomic target to a clinically important drug class. Influenza neuraminidase structures similarly enabled transition-state-inspired inhibitors [27],[28]. Moreover, work on severe acute respiratory syndrome coronavirus 2 (SARS-CoV-2) main protease, HIV-1 capsid, respiratory syncytial virus (RSV) prefusion F, and coronavirus spike extended this logic [29]–[34]. These successes required more than a structure: Kinetics, thermodynamics, resistance, antigenicity, manufacturing, and clinical testing determined whether a molecular model was actionable.

The available evidence is taxonomically uneven. HIV-1 and SARS-CoV-2 dominate because they have unusually dense structural, biochemical, simulation, and clinical datasets. Influenza virus, hepatitis B virus (HBV), human papillomavirus (HPV), Ebola virus, adenovirus, bacteriophages, and non-enveloped enteric viruses provide essential tests of whether a proposed principle remains valid across differences in genome type, envelope architecture, receptor usage, and environmental exposure [5],[11],[35]–[45].

Throughout this review, broad claims are separated from conclusions that are specific to a single protein, strain, experimental construct, or viral family. Rather than providing a technique-by-technique inventory, we critically integrate experimental, computational, and biochemical evidence to assess how reliably molecular-scale observations can be connected to microbiologically and clinically relevant phenotypes. This perspective guides the organization of the review, from comparing complementary methods and experimentally constrained workflows to evaluating biophysical principles across protein classes, viral families, membrane-associated processes, and interaction networks. We then consider how these mechanistic insights translate into antiviral development and vaccine design, including virus-like particles (VLPs), before addressing the methodological, microbiological, and artificial intelligence (AI)-related limitations that currently restrict interpretation and generalization. Across these topics, emphasis is placed on the questions that each approach can answer, the evidence required to validate mechanistic models, and the boundaries beyond which extrapolation is not justified.

## 2. Biophysical approaches to viral proteins

### 2.1. Experimental methods

#### 2.1.1. Nuclear magnetic resonance spectroscopy

NMR is most informative when the relevant question concerns an ensemble rather than a single dominant structure. Chemical shifts, relaxation, residual dipolar couplings, paramagnetic effects, and exchange measurements can resolve local order, low-population states, and interconversion processes spanning timescales from picoseconds to seconds [46],[47]. Studies on HIV-1 trans-activator of transcription (Tat), influenza nucleoproteins, hepatitis C virus nonstructural proteins, and coronavirus accessory proteins illustrate how disorder and transient binding can regulate host-factor recruitment [48]–[59]. The interpretation is nevertheless conditional. Molecular size, spectral overlap, isotope-labeling requirements, exchange broadening, and the use of truncated constructs can complicate state assignment; a chemical-shift perturbation alone does not establish a functional binding mechanism. NMR-derived ensembles are therefore strongest when populations and kinetics are validated by biochemical or mutational assays and by orthogonal structural or dynamic measurements [46],[60],[61].

#### 2.1.2. Cryo-Electron Microscopy

Cryo-EM provides high-resolution architectures of large viral assemblies and can separate recurrent conformational classes [11],[35],[62]–[65]. Its principal contribution is not simply high nominal resolution, but the ability to relate subunit organization, ligand occupancy, and structural heterogeneity in glycoproteins, capsids, and replication complexes. However, local resolution is often much poorer in flexible loops, glycans, membrane-proximal regions, and low-occupancy states. Vitrification, preferred orientation, particle selection, and three-dimensional classification can reshape a continuous conformational distribution into apparently discrete states [66]. Time-resolved experiments enrich intermediates but do not automatically establish their temporal order or kinetic connectivity [67]. Cryo-EM models should therefore be evaluated against biochemical stoichiometry, NMR or single-molecule dynamics, and simulations that reproduce the density without overfitting [60],[68],[69].

#### 2.1.3. Atomic Force Microscopy

AFM supplies an observable that structural methods do not: The response of a virion, capsid, or membrane to a defined mechanical perturbation [15],[70]. Measurements on bacteriophage ΦKZ and HIV-1 link shell stiffness and deformability to genome protection, nuclear entry, uncoating, and infectivity [36],[71]–[74]. High-speed AFM can additionally follow surface rearrangements [15],[70],[75]. Mechanical values are not intrinsic constants, however. Immobilization chemistry, hydration, tip radius, loading rate, indentation depth, and the constitutive model used to fit force curves can change the apparent modulus or rupture force. Consequently, AFM is most persuasive when relative changes are reproduced across different loading conditions and interpreted in conjunction with structural models, simulated indentation, and infectivity assays [71],[72],[76].

#### 2.1.4. Isothermal titration calorimetry and differential scanning calorimetry

Isothermal titration calorimetry (ITC) and differential scanning calorimetry (DSC) quantify the energetic consequences of binding and unfolding [16],[17]. ITC can determine affinity, stoichiometry, and binding enthalpy, while DSC reports cooperative thermal transitions and ligand- or mutation-dependent stability. Thermodynamic measurements have provided mechanistic insight into viral protein–ligand interactions and conformational changes associated with viral fusion [77]. Their mechanistic reach is also bounded. ITC requires an appropriate concentration window, accurate determination of active concentrations, matched buffers, and explicit treatment of proton uptake, linked equilibria, and aggregation; the entropy term is inferred rather than measured independently. DSC transitions may combine unfolding, oligomer dissociation, and irreversible aggregation [17]. Calorimetry therefore can discriminate energetic models but rarely identifies a binding pose or kinetic pathway without structural, kinetic, and mutational evidence.

#### 2.1.5. Critical comparison and method selection

Method selection should follow the observable required, the lifetime of the state, and the biological scale of the claim. NMR resolves local dynamics and exchange, cryo-EM defines architectures and occupancies, AFM measures mechanical response, and calorimetry quantifies binding or unfolding energetics. None is a universal reference method. Table 1 therefore compares practical resolution, sample requirements, throughput, cost, strengths, and major sources of bias rather than ranking methods according to a single performance metric. Cross-validation is most informative when two techniques report on the same quantity or on explicitly linked quantities. Cryo-EM structures can provide starting coordinates for NMR-restrained ensembles; AFM force curves can be compared with simulated indentation; and ITC affinities can be tested by alchemical or potential-of-mean-force calculations and targeted mutations [16],[61],[68],[71],[78],[79]. Agreement by visual inspection is insufficient. The model should predict an independent observable, and disagreement should be used to identify missing states, sample perturbation, or an inadequate physical representation. Figure 1 uses HIV-1 because its life cycle provides experimentally tractable examples for each method discussed: Envelope conformations, Tat-ribonucleic acid (RNA) dynamics, capsid mechanics, and protease thermodynamics. These examples illustrate the complementarity of the different observables, while their broader relevance across viral families is considered in Section 3.4.

**Table 1.** Practical comparison of experimental biophysical approaches applied to viral proteins.

Method	Primary observable and practical resolution	Sample, throughput, and cost	Main strengths	Main limitations and recommended cross-validation
NMR spectroscopy	Residue-specific chemical environment, distances, order parameters, and exchange from picoseconds to seconds; highest detail for small to medium proteins, with specialized approaches for larger assemblies.	Typical milligram quantities; isotope labeling is often required; moderate throughput; high instrument and analysis cost.	Direct access to disorder, low-population states, and kinetics in solution or membranes.	Size, overlap, labeling, and exchange broadening; validate ensembles with mutagenesis, binding or kinetic assays, cryo-EM, or single-molecule data [46]–[54],[80].
Cryo-EM	Three-dimensional density maps; approximately 2-4 Å in well-ordered regions, often lower locally; particle classes can reveal recurrent heterogeneity.	Microgram-scale purified material but demanding specimen optimization; low to moderate throughput; very high capital and computational cost.	Large complexes, native-like assemblies, ligand occupancy, and multiple structural states.	Vitrification, orientation, local-resolution, classification, and model-bias effects; validate with biochemistry, NMR, smFRET, or simulations against withheld density [11],[35],[60],[62]–[69].
AFM	Nanometer topography and pico- to nanonewton force response; high-speed imaging can reach millisecond-to-second dynamics.	Surface-immobilized particles or membranes; moderate throughput; moderate instrument cost but substantial calibration and analysis.	Direct mechanics under hydrated conditions; single-particle heterogeneity.	Tip geometry, loading rate, immobilization, and mechanical model dependence; reproduce across conditions and compare with simulated deformation and infectivity [15],[36],[70]–[76].
ITC	Affinity, stoichiometry, and binding enthalpy over an experiment-dependent concentration window.	Relatively high amounts of homogeneous sample; low throughput; moderate instrument cost.	Label-free, solution-phase thermodynamic discrimination of binding models.	Concentration errors, buffer protonation, aggregation, linked equilibria, and limited kinetic information; pair with kinetics, structures, and mutagenesis [16],[77].
DSC	Heat-capacity profile, transition temperature, and cooperative unfolding or dissociation.	Relatively high sample requirement; low to moderate throughput; moderate cost.	Global stability and ligand- or mutation-dependent thermal transitions.	Irreversibility and overlap of unfolding, oligomer dissociation, or aggregation; pair with spectroscopy, scattering, and functional assays [17],[81].

Abbreviations: NMR, Nuclear magnetic resonance; cryo-EM, Cryo-electron microscopy; smFRET, Single-molecule Förster resonance energy transfer; AFM, Atomic force microscopy; ITC, Isothermal titration calorimetry; DSC, Differential scanning calorimetry.

**Figure 1.**
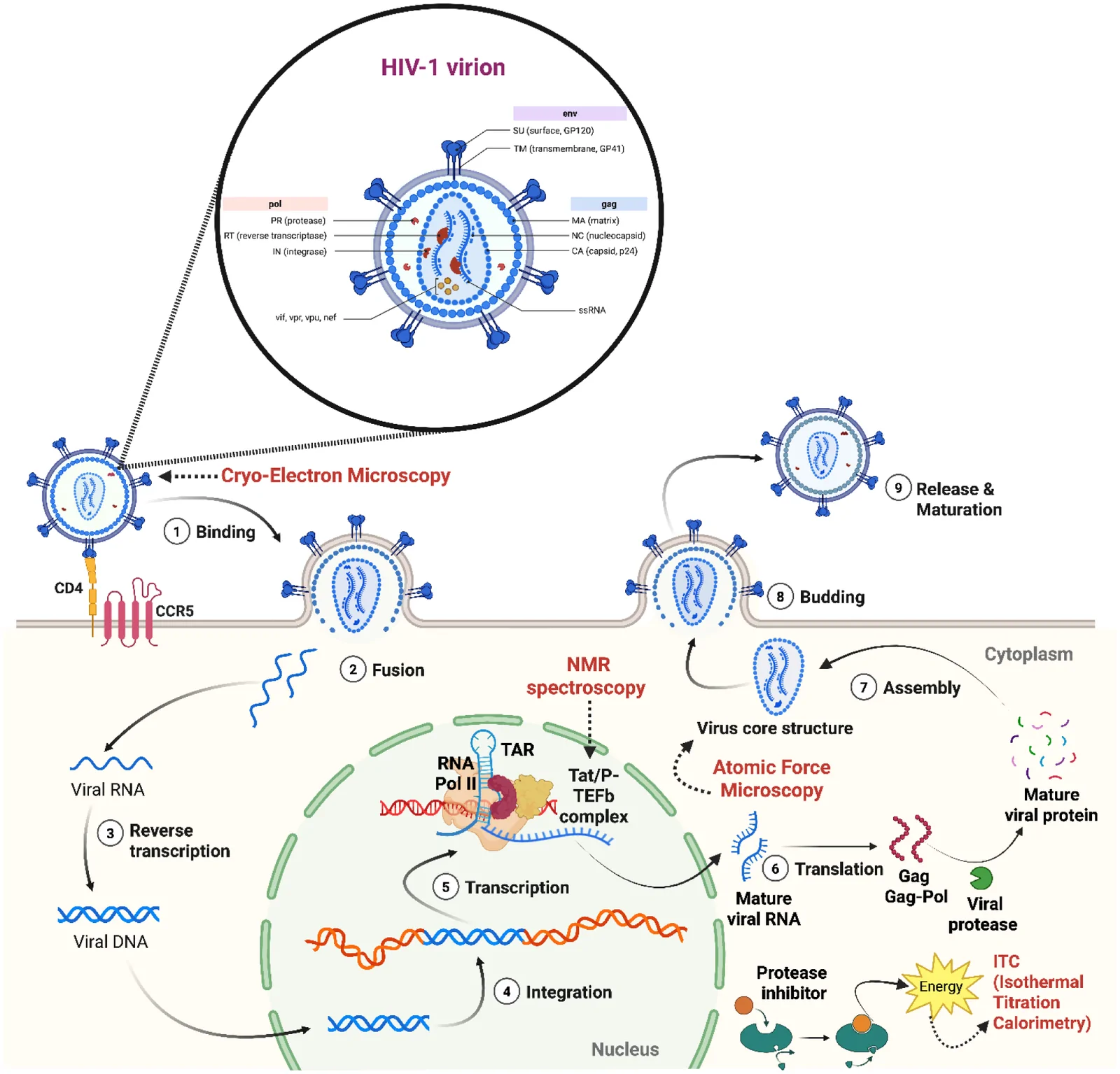
HIV-1 as an illustrative example of method selection across a viral life cycle. Cryogenic electron microscopy resolves virion and envelope architectures; nuclear magnetic resonance spectroscopy characterizes dynamic Tat-RNA and other transient interactions; atomic force microscopy probes capsid mechanics; and isothermal titration calorimetry quantifies protease-inhibitor energetics. HIV-1 is selected because these complementary observables are available within one extensively studied system.

### 2.2. Computational methods

All-atom MD and coarse-grained MD are complementary resolutions within a simulation hierarchy rather than separate methodological families. Atomistic models retain local chemistry but are limited in size and sampling; coarse-grained models integrate over selected degrees of freedom to access larger assemblies and longer timescales. Structural bioinformatics and AI provide structural and statistical priors, but they do not generate physical trajectories unless coupled to an explicit dynamical model.

#### 2.2.1. All-atom MD simulations

All-atom MD represents each atom and propagates atomic motions according to an empirical force field. The force field, protonation states, ion parameters, glycan composition, membrane lipid mixture, water model, and starting structure are therefore part of the model definition and can materially affect the resulting ensemble. Landmark developments in biomolecular force fields and membrane simulation established the basis for modern viral-protein calculations [82]–[86]. For viral envelopes and fusion proteins, membrane composition and glycosylation can substantially shift conformational populations; for enzymes, catalytic ions and protonation states require particular care. A trajectory is interpretable only within the thermodynamic state and chemical representation that are simulated. Conventional trajectories often undersample activation barriers. Replica-exchange MD, metadynamics, and accelerated MD can broaden sampling, whereas umbrella sampling and related methods can quantify relative free energies of different states or binding configurations [87]–[89]. These approaches introduce new assumptions: Replica ladders, collective variables, bias deposition, restraint pathways, and reweighting can determine the result. Convergence should be assessed with independent replicas, time-resolved observables, state-population stability, and uncertainty estimates; free-energy estimates should report overlap and statistical error, for example through multistate estimators [90]. Reproducibility also requires deposited structures, parameters, software versions, random seeds, and analysis definitions. Applications to influenza hemagglutinin, HIV-1 envelope (Env), SARS-CoV-2 spike, and HIV-1 protease show the value of this discipline when simulations explain an experimental discrepancy or make prospective predictions [64],[91]–[98]. Simulations are less informative when one trajectory is presented as a complete pathway, when only a final root-mean-square deviation is reported, or when agreement is judged against the starting structure. Direct comparison with NMR populations, cryo-EM densities, single-molecule Förster resonance energy transfer (smFRET) distributions, binding thermodynamics, enzyme kinetics, or mutational effects is the appropriate standard [61],[68],[79].

#### 2.2.2. Coarse-Grained MD simulations

Coarse-grained MD is a lower-resolution form of MD in which groups of atoms are represented by interaction sites. It is not universally derived from all-atom MD: Models may be parameterized top-down against thermodynamic observables, bottom-up through force matching, or by hybrid strategies [82],[85]. The Martini framework of Marrink, Tieleman, and colleagues enabled transferable simulations of lipids and biomolecules, while multiscale approaches from Bond, Voth, and others established strategies for connecting atomistic and mesoscopic descriptions [82],[83],[85]. The gain in system size and timescale is achieved at the cost of smoother energy landscapes, reduced chemical specificity, and kinetics that may not map directly to physical time. Coarse-grained MD is particularly useful for capsid assembly, membrane remodeling, genome-capsid coupling, and whole-envelope organization [5],[99]–[102]. Parameterization and validation should match the intended question: A model fitted to equilibrium partitioning may reproduce morphology but not assembly kinetics, while a structure-based model may preserve a known lattice yet miss alternative polymorphs. Backmapping to all-atom resolution and comparison with cryo-EM, AFM, scattering, or assembly kinetics can reveal whether a mesoscale mechanism remains valid after restoration of chemical detail.

#### 2.2.3. Structural bioinformatics

Structural bioinformatics compares sequences, structures, motifs, coevolution, surfaces, and pockets across many viral proteins [23],[103]–[105]. Its strength is scale: Conservation and covariation can prioritize residues for mutagenesis, identify candidate epitopes, and place a new structure within its evolutionary context. Its main limitation is inferential. Database composition, sequence redundancy, alignment quality, and phylogenetic non-independence can make a motif appear more general than it actually is. Moreover, static structure comparison does not establish state populations or kinetics. Thus, bioinformatic predictions should be treated as testable priors for biochemical assays, structural analysis, or simulations, rather than as mechanistic endpoints.

#### 2.2.4. Validation, reproducibility, and integrative computational modeling

Computational models gain mechanistic credibility when they reproduce an experimental observable that was not used in model construction. Cryo-EM density, NMR chemical shifts or distances, smFRET distributions, AFM force-response curves, calorimetric affinities, enzyme kinetics, and resistance phenotypes provide complementary tests [61],[68],[79]. Experimentally restrained simulations can reduce underdetermination, but restraints do not remove model bias: Excessive weighting can force agreement while hiding an incorrect force field or a missing state. A minimum reporting standard should include the modeled biological state, representation and parameter set, system composition, equilibration protocol, total sampling across replicas, convergence diagnostics, uncertainty, and the data reserved for validation. All-atom MD, coarse-grained MD, bioinformatics, and AI-assisted models answer different questions; Table 2 compares their relevant scales and validation requirements. This framework avoids treating atomistic and coarse-grained approaches as competing methods and clarifies how and when information can be transferred across resolutions.

**Table 2.** Comparison of computational approaches, accessible scales, and validation requirements in viral protein biophysics.

Approach	Representation and primary question	Typical accessible scale and cost	Main strengths	Principal limitations and validation
All-atom MD	Atomic coordinates under an empirical force field; local interactions, conformational exchange, membrane coupling, and ligand binding.	Usually nanoseconds to microseconds for systems from proteins to large membrane complexes; high to very high computational cost.	Chemical detail, hydration, ion and glycan interactions, and direct comparison with residue-level observables.	Force-field and system-construction bias, limited sampling, and protonation uncertainty; validate with NMR, cryo-EM, smFRET, calorimetry, kinetics, and independent replicas [22],[64],[79],[84],[86],[91]–[98],[106].
Coarse-grained MD	Groups of atoms represented as beads; assembly, membrane remodeling, capsid mechanics, and mesoscale organization.	Microseconds to milliseconds and large assemblies; moderate to high cost depending on system size.	Access to collective processes and system sizes that are impractical atomistically.	Reduced chemical specificity, model-dependent kinetics, and parameter transferability; validate against structures, scattering, AFM, assembly kinetics, and backmapped simulations [5],[82],[83],[85],[99]–[102].
Structural bioinformatics	Sequence and structure comparison, conservation, coevolution, epitope and pocket prioritization.	Proteome- to database-scale; low to moderate cost and high throughput.	Broad evolutionary context and rapid hypothesis generation.	Sampling and phylogenetic bias; static predictions do not establish dynamics or function; validate by prospective mutagenesis and experimental structure or binding [23],[103]–[105].
Enhanced-sampling and free-energy MD	Biased or replica-based sampling of rare transitions and thermodynamic differences.	System- and method-dependent; often very high aggregate cost.	Quantitative state or binding free energies and access to otherwise rare events.	Collective-variable, pathway, overlap, and reweighting dependence; report convergence, replicas, uncertainty, and prospective tests [87]–[90].
AI and hybrid structural modeling	Predicted coordinates or latent conformational representations integrated with experimental data.	Rapid prediction to compute-intensive heterogeneous reconstruction; variable cost.	Coverage of unsolved proteins, model completion, and organization of heterogeneous datasets.	Training-set bias, uncertain alternative states, and no intrinsic functional proof; validate with experimental restraints and withheld functional data [107]–[111].

Abbreviations: MD, molecular dynamics; NMR, nuclear magnetic resonance; cryo-EM, cryogenic electron microscopy; smFRET, single-molecule Förster resonance energy transfer; AFM, atomic force microscopy; AI, artificial intelligence.

### 2.3. Biochemical perspectives on viral proteins

#### 2.3.1. Catalytic mechanisms and inhibitor response

Viral proteases, polymerases, and neuraminidases illustrate how biochemical kinetics constrains structural interpretation. Active-site geometry and transition-state mimicry guided the development of HIV-1 protease and influenza neuraminidase inhibitors [25]–[28], whereas resistance studies show that altered dynamics or substrate recognition can reduce efficacy without abolishing catalysis [98],[112]. Enzyme structures should therefore be interpreted together with catalytic efficiency, inhibition constants, drug residence time, and phenotypic susceptibility.

#### 2.3.2. Post-translational modifications and host interactions

Glycosylation, phosphorylation, ubiquitination, and related modifications alter folding, trafficking, receptor binding, and immune recognition [113]–[117]. SARS-CoV-2 spike glycosylation illustrates a central limitation: Occupancy and glycan processing depend on cell type and expression system, so a recombinant glycoprofile may not fully recapitulate that of the native virion [113],[118],[119]. Modification maps become mechanistic only when site-specific perturbation changes a defined biochemical or infection phenotype.

#### 2.3.3. Biochemical reconstitution of assembly and replication

Reconstitution reduces a viral process to controlled components and is especially valuable for capsid assembly and polymerase complexes. Controlled biochemical and structural studies of HBV core proteins and influenza polymerase have clarified capsid assembly, polymerase organization, and host-factor dependence [37],[38],[120],[121]. Its strength lies in causal control, whereas its main limitation is the absence of crowding, membranes, compartmentalization, and host factors. Concentrations and stoichiometries should therefore be compared with those in the cellular environment before an *in vitro* rate-limiting step is considered dominant during infection.

#### 2.3.4. Environmental regulation and transient intermediates

pH, ionic strength, redox state, proteolysis, and membrane composition can switch viral proteins between functional states. Ebola glycoprotein provides examples in which acidic pH, sequence variation, proteolytic priming, and receptor-independent membrane binding alter fusion competence [39],[40],[122]. Because triggering is often asynchronous and irreversible, apparent intermediates may reflect kinetic trapping or sample preparation. Time-resolved triggering, reversibility tests, and orthogonal structural or kinetic measurements are needed to establish the order of events along the pathway.

#### 2.3.5. Biochemical validation of therapeutic targets

Docking, structural models, and MD can prioritize antiviral compounds, but target engagement and antiviral activity must be demonstrated independently. Studies of SARS-CoV-2 protease inhibitors and broad-spectrum peptides illustrate the required progression from binding or enzymatic inhibition to cell-based activity and resistance testing [29],[33],[78],[123],[124]. Cytotoxicity, aggregation, assay interference, and limited cellular exposure can explain discordance between biophysical affinity and antiviral activity.

#### 2.3.6. Biochemical constraints for integrative models

Biochemical measurements can provide causal tests that connect structure and simulation to infection. Mutations can test a predicted interface, kinetic assays can discriminate between binding and catalysis, and reconstitution can identify missing cofactors. The purpose is not to force all datasets into a single model, but to determine whether the model predicts function under defined conditions. Table 3 summarizes these biochemical domains and their principal interpretive limits; in Section 2.4, we develop the cross-method workflow.

**Table 3.** Biochemical mechanisms, functional tests, and principal interpretive limitations.

Biochemical domain	Question and measurement	Functional inference	Principal limitation	Representative studies
Catalytic functions of viral enzymes	Mechanistic analysis of catalysis, substrate recognition, and inhibitor binding.	Controls proteolytic processing, genome replication, and drug response.	A structure alone does not establish catalytic rate, residence time, or resistance phenotype.	HIV‑1 protease [125]; neuraminidase inhibitors [126]; HIV-1 protease resistance [98],[112].
Post‑translational modifications	Mapping of glycosylation, phosphorylation, ubiquitination, acetylation, and SUMOylation.	Regulates entry, immune evasion, and the assembly of the replication complex.	Occupancy and processing depend on cell type, expression system, and viral background.	SARS-CoV-2 S glycosylation [113],[118],[119]; NP phosphorylation [117]; NP acetylation [127]; Vif ubiquitination [115],[128]; NS1 SUMOylation [129].
Biochemical reconstitution systems	*In vitro* reconstruction of viral processes.	Dissects assembly, fusion, and replication steps.	Simplified concentrations, stoichiometry, and missing host factors can alter pathways.	FluPol assembly [120],[121]; HBV core encapsidation [37]; capsid modulators [38].
Environmental and chemical modulation	Effects of pH, ions, and chemical cues on protein conformation.	Triggers fusion activation and conformational switching.	Triggered states may be asynchronous, irreversible, or created by trapping conditions.	Ebola GP pH response [122]; A82V fusion enhancement [39]; membrane binding [40].
Integration with structural and computational methods	Combining biochemical data with cryo-EM, all-atom MD, and tomography.	Links kinetics and mutations to structural dynamics.	Restraints can force agreement; validation should use an observable not used in model construction.	Hybrid biochemical-MD studies [78],[123],[130]; HIV-1 protease dynamics [98],[112].
Immune recognition and evasion	Analysis of epitopes, glycan shields.	Shapes antibody escape and T‑cell recognition.	Epitope exposure *in vitro* may not predict neutralization, processing, or population escape.	Glycan-gate dynamics [119]; conserved epitopes [131].
Validation of antiviral targets	*In vitro* screening of inhibitors and binding kinetics.	Confirms the druggability of viral enzymes and structural proteins.	Binding or enzyme inhibition may reflect assay interference, aggregation, or poor cellular exposure.	3CLpro inhibitors [29],[33],[78],[123]; HIV-1 capsid inhibition [30].
Clinically validated antiviral and vaccine targets	Integration of structural, binding, enzymatic, and cell-based evidence.	Connects molecular mechanism to therapeutic and vaccine performance.	Clinical actionability requires manufacturing, safety, resistance, immunology, and efficacy evidence.	HIV-1 capsid inhibition [30]; SARS-CoV-2 Mpro inhibition [29],[33]; prefusion-stabilized immunogens [31],[34],[132].

Abbreviations: HIV-1, Human immunodeficiency virus type 1; SARS-CoV-2, Severe acute respiratory syndrome coronavirus 2; S, Spike protein; NP, Nucleoprotein; Vif, Viral infectivity factor; NS1, Nonstructural protein 1; FluPol, Influenza virus RNA polymerase; HBV, Hepatitis B virus; GP, Glycoprotein; cryo-EM, Cryogenic electron microscopy; MD, Molecular dynamics; 3CLpro, 3C-like protease; Mpro, Main protease.

### 2.4. Integrative workflows across experimental and computational scales

Integrative viral protein biophysics begins with a biological question at a defined stage of infection and with a specified microbiological phenotype. Host range may require receptor-binding and fusion measurements; environmental persistence may require capsid stability and inactivation kinetics; immune escape may require structural mapping, binding thermodynamics, neutralization, and sequence surveillance; resistance requires target engagement, enzyme or assembly assays, and phenotypic susceptibility. The method is selected only after the observable and decision criterion are defined. The SARS-CoV-2 spike illustrates an iterative workflow. Cryo-EM defined prefusion architecture and receptor-accessible states of the receptor-binding domain [133],[134]; all-atom MD predicted glycan-dependent changes in those populations [135]; binding measurements and structure-based engineering were then used to test whether the predicted states altered receptor engagement or antigen stability [136]. The inference is strongest when simulation and experiment share an observable. It is weaker when a structural model is used to infer transmissibility without measurements of entry, replication, or epidemiological fitness. Other pairings test different levels of mechanism. NMR restraints can refine flexible regions that are poorly resolved in cryo-EM; AFM measurements and multiscale simulations can connect capsid deformation to uncoating or nuclear-pore transit; and calorimetry, free-energy calculations, and mutational assays can separate altered affinity from altered conformational populations [16],[61],[68],[71],[72],[78],[79]. AI-predicted structures can enter the same loop, but confidence scores are not substitutes for state-specific experimental restraints. Figure 2 formalizes this sequence from biological question to orthogonal validation; each arrow represents a falsifiable comparison. Disagreement with independent measurements should prompt model revision or an explicit statement of the remaining uncertainty.

## 3. Case studies

### 3.1. Capsid proteins

Capsids expose conserved physical principles and virus-specific solutions. In HBV, core-protein assembly, capsid modulation, and drug-resistant assembly phenotypes have been investigated using complementary biochemical, biophysical, and molecular simulation approaches [37],[137]–[139]. HIV-1 adds a mechanically active, pleomorphic core whose curvature and elasticity influence nuclear entry and uncoating [71],[72],[102],[140]. Bacteriophages and non-enveloped viruses emphasize genome pressure, shell reinforcement, and extracellular stability [5],[36],[141]. Across these systems, electrostatics, hydrophobic contacts, and conformational entropy contribute to assembly, but their relative importance and rate-limiting steps differ. The microbiological consequence depends on context. Increased shell stiffness may protect the genome outside the host yet impede uncoating, whereas greater elasticity may facilitate passage through a pore but reduce extracellular durability. Capsid-targeting antivirals exploit these assembly and stability constraints in HBV and HIV-1, but resistance can arise through altered interfaces or alternative assembly pathways [30],[137]–[139]. Thus, morphology or stiffness cannot predict fitness; assembly kinetics, genome packaging, uncoating, and infectivity must be measured in the same genetic background.

**Figure 2.**
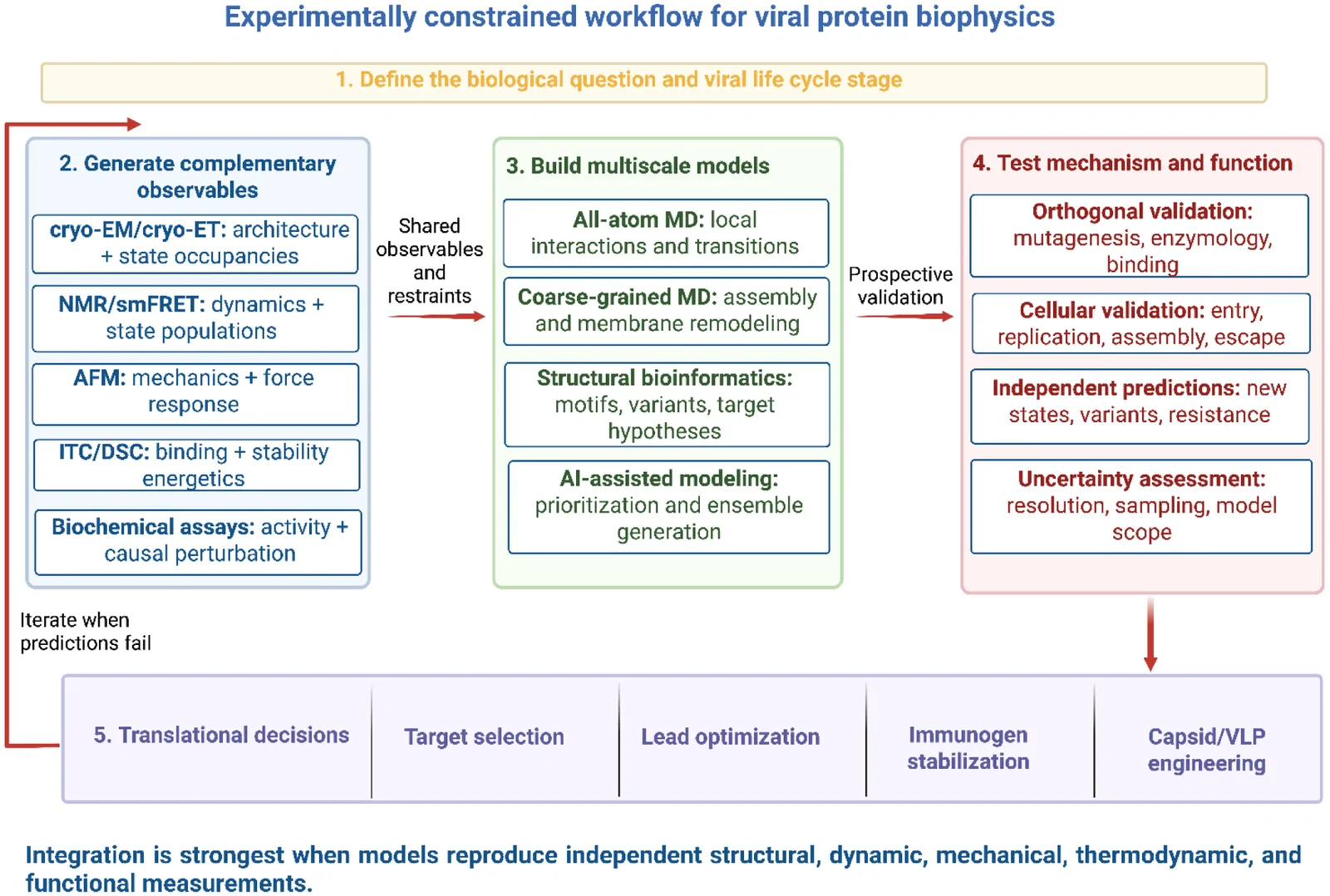
Experimentally constrained workflow for integrative viral protein biophysics. A question is defined at a viral life-cycle stage and linked to a microbiological or translational endpoint, such as tropism, environmental persistence, immune escape, resistance, target selection, or immunogen design. Cryogenic electron microscopy, nuclear magnetic resonance spectroscopy, atomic force microscopy, calorimetry, and biochemical assays provide structural, dynamic, mechanical, energetic, and functional observables. All-atom molecular dynamics (MD), coarse-grained MD, structural bioinformatics, and artificial intelligence (AI)-assisted models are constrained and tested against these data. Arrows indicate the progression of the workflow, while links between model predictions and independent validation represent falsifiable comparisons with experimental or biological observables. Independent cellular, virological, surveillance, or clinical evidence closes the loop. Iteration is required when predictions fail to reproduce independent measurements.

### 3.2. Fusion proteins

Fusion proteins share a metastable prefusion state and a refolding pathway that couples receptor or pH sensing to membrane merger, but the triggers and accessible intermediates are not interchangeable. Cryo-EM, NMR, spectroscopy, biochemical triggering, and MD have resolved receptor-binding-domain motion in coronavirus spike, helical-bundle formation in HIV-1 glycoprotein 41 (gp41), pH switches in influenza hemagglutinin, and receptor- and protease-dependent activation of Ebola glycoprotein [35],[39],[40],[62],[77],[93],[142]–[147]. These studies support a general energy-landscape framework while showing that family-specific cleavage, glycosylation, membrane composition, and receptor usage determine which intermediate controls entry. These molecular differences can be linked to host range and transmission-relevant phenotypes only with additional evidence. Receptor affinity, protease compatibility, prefusion-state occupancy, and membrane fusion efficiency can affect cell tropism, but population spread also depends on replication, immune history, and exposure. Variant studies should therefore combine structural or simulation results with entry assays, primary-cell replication, neutralization, and epidemiological comparisons rather than equating tighter binding with greater transmissibility.

### 3.3. Accessory and regulatory proteins

Accessory and regulatory proteins are less structurally conserved and more dependent on cellular context. Influenza nonstructural protein 1 (NS1), HIV-1 viral infectivity factor (Vif), and coronavirus open reading frames 6 and 8 (ORF6 and ORF8) use flexible motifs, post-translational modifications, and multivalent host interactions to suppress sensing or restriction pathways [55]–[57],[148]–[153]. Structural or bioinformatic identification of a motif is therefore only the first level of evidence. Isoform expression, stoichiometry, subcellular localization, cell type, and viral genetic background can change the phenotype, as illustrated by apparently conflicting results for coronavirus accessory proteins [56],[153]. Mechanistic claims require perturbation in infection-relevant systems, not only isolated binding measurements. This context dependence limits cross-family generalization and enhances microbiological relevance. Accessory-protein variation can shift innate immune antagonism, cell-type permissiveness, or persistence without changing virion architecture. Integrating structural data with proteomics, single-cell infection, and comparative viral genetics is therefore more informative than attempting to assign a universal fold-function rule.

### 3.4. Cross-family generalization and microbiological phenotypes

A biophysical principle should be considered general only when it is supported in phylogenetically and structurally distinct systems. Regulated conformational transitions during viral entry are observed across coronaviruses, orthomyxoviruses, retroviruses, and filoviruses, whereas the precise trigger and kinetic bottleneck are family-specific. Capsid mechanics influences infectivity in retroviruses and bacteriophages, but the mechanical constraint differs between nuclear-pore transit, genome packaging, and extracellular persistence. Thus, Table 4 maps each principle to a microbiological phenotype and states the limits of the supporting evidence. HIV-1 and SARS-CoV-2 remain prominent because they permit unusually complete workflows from structure to clinical outcome. They are treated here as well-characterized exemplars rather than proxies for overall viral diversity: HBV and HPV VLPs test assembly and antigen display; influenza and Ebola test distinct fusion triggers; adenovirus and bacteriophages test non-enveloped mechanics and environmental exposure. Claims that remain supported only in a data-rich exemplar are identified as hypotheses for broader testing rather than established general principles.

**Table 4.** Cross-family evidence linking biophysical determinants to microbiological phenotypes.

Viral system	Biophysical determinant	Microbiological phenotype	Integrated evidence	Scope and limitation
HBV	Core-protein affinity, nucleic-acid coupling, and assembly kinetics.	Genome packaging, virion production, and susceptibility to capsid assembly modulators.	Reconstitution, calorimetry, light scattering, structural analysis, and simulation [37],[38],[137]–[139],[154].	Strong mechanistic evidence, but *in vitro* concentrations and host-factor absence can shift pathways.
HIV-1	Capsid curvature and elasticity; Env and protease conformational dynamics.	Nuclear entry, uncoating, immune recognition, and drug resistance.	AFM, cryo-EM, calorimetry, MD, and virology [30],[64],[71],[72],[77],[98],[112].	Unusually dense evidence; mechanisms should not be extrapolated to unrelated capsid architectures without testing.
Influenza A virus	pH-triggered hemagglutinin refolding and neuraminidase active-site geometry.	Cell entry, host adaptation, antigenic change, and antiviral susceptibility.	Structures, spectroscopy, MD, enzymology, and resistance analysis [27],[28],[35],[91]–[93].	Subtype and host differences alter activation and receptor context.
HPV	L1 self-assembly and conformational epitope display.	Particle immunogenicity and vaccine antigen quality.	Structural assembly and immunogenicity of VLPs [41].	Licensed VLP success supports the principle; genotype breadth and manufacturing remain platform-specific.
SARS-CoV-2	Spike conformational populations, glycan shielding, and protease activation.	Tropism, entry efficiency, immune escape, and vaccine-antigen performance.	Cryo-EM, all-atom MD, binding, neutralization, and surveillance [62],[97],[118],[133]–[136],[155].	Binding or RBD opening alone does not establish transmissibility or clinical severity.
Ebola virus	pH-, protease-, receptor-, and sequence-dependent glycoprotein activation.	Cell tropism and fusion competence.	Structural, biochemical, membrane-binding, and functional studies [39],[40],[122],[147].	Trapped intermediates and simplified membranes may not reproduce endosomal kinetics.
Bacteriophages and non-enveloped viruses	Shell mechanics, genome pressure, and exposure of membrane-active capsid elements.	Environmental persistence, genome delivery, and infectivity.	AFM, structural biology, biochemical assays, and simulation [5],[36],[43],[65],[141].	The relevant mechanical load differs from that in enveloped or nuclear-entering viruses.

Abbreviations: HBV, Hepatitis B virus; HIV-1, Human immunodeficiency virus type 1; Env, Envelope glycoprotein; AFM, Atomic force microscopy; cryo-EM, Cryogenic electron microscopy; MD, Molecular dynamics; HPV, Human papillomavirus; VLP, Virus-like particle; SARS-CoV-2, Severe acute respiratory syndrome coronavirus 2; RBD, Receptor-binding domain.

## 4. Molecular interactions

### 4.1. Protein-membrane interactions

Protein-membrane interactions determine whether receptor engagement is converted into entry, budding, or genome release. Spectroscopy identifies membrane-induced secondary-structure changes in fusion peptides, AFM visualizes local deformation, and all-atom or coarse-grained MD simulations test how lipid composition, curvature, and peptide insertion alter the free-energy landscape [15],[99],[156]–[160]. Similar logic applies to non-enveloped viruses, whose capsids expose membrane-active elements during uncoating [43],[65],[141]. These mechanisms can influence cell tropism because receptor abundance alone is insufficient when a viral protein is incompatible with the lipid composition, protease environment, or endosomal pH of a target cell. The evidence must also distinguish membrane binding from productive fusion. Peptides can insert into simplified bilayers at concentrations or lipid compositions not reached during infection, and supported membranes can suppress curvature and asymmetry. Claims about productive fusion should be validated using infection-relevant systems, ideally including full-length proteins or virions, relevant proteolytic activation, lipid mixtures that approximate those of the target organelle, and assays of content mixing or infectivity. These constraints are particularly important when translating membrane-active peptides into fusion inhibitors [161]–[163]. Table 5 summarizes these protein-membrane interaction mechanisms.

**Table 5.** Summary of protein-membrane interactions.

Biological process	Techniques	Key findings	References
Fusion peptide insertion and membrane destabilization	FTIR, CD spectroscopy.	Coil-to-helix transitions; structural plasticity enabling adaptation to lipid environments.	[156]–[158]
Membrane curvature and deformation	AFM.	Local bilayer deformation associated with viral entry, including influenza HA-mediated processes.	[15],[70],[160]
Amphipathic helix insertion and curvature generation	All-atom MD simulations.	Atomistic details of lipid rearrangement and pore-forming curvature.	[157]
Capsid–membrane interactions in non-enveloped viruses	Structural biology, functional assays.	Exposure of hydrophobic loops facilitating genome release.	[43],[65],[141]
Fusion inhibition	Antiviral peptide design.	Coronin-derived peptides and measles fusion inhibitors blocking peptide insertion.	[161],[162]
Biotechnological applications	Nanotechnology.	Viral peptides used to engineer synthetic vesicles and delivery systems.	[164]

Abbreviations: FTIR, Fourier transform infrared spectroscopy; CD, Circular dichroism; AFM, Atomic force microscopy; HA, hemagglutinin; MD, molecular dynamics.

### 4.2. Protein-protein interactions

Protein-protein interactions control capsid assembly, polymerase activity, and recruitment or degradation of host factors. Calorimetry and reconstitution quantify assembly cooperativity; cryo-EM resolves multimeric polymerase states; and mutational or cellular assays test whether an interface is required during infection [5],[121],[150],[165]–[169]. The central distinction is between physical interaction and functional dependence. High affinity *in vitro* may be irrelevant at cellular stoichiometry, whereas weak multivalent contacts can dominate in condensates or membrane-associated complexes. These interactions also connect molecular variation to microbiological outcomes. Mutations at receptor, polymerase-cofactor, or restriction-factor interfaces can alter host range, replication efficiency, immune escape, or drug resistance, but the effect may be compensated by changes elsewhere in the viral genome. Cross-family comparison and experiments that account for epistasis are therefore necessary before an interface is designated a broadly conserved vulnerability. Table 6 summarizes representative interactions and the evidence used to assign their functional relevance.

**Table 6.** Summary of protein-protein interactions.

Biological process	Techniques	Key findings	References
Cooperative capsid assembly	ITC; cryo-EM and mutational analysis.	Enthalpic/entropic contributions; sensitivity to mutations and environmental conditions.	[169],[170]
Capsid assembly dynamics	All-atom MD and metadynamics.	Identification of rare intermediates and kinetic traps.	[5]
Multimeric polymerase complexes	Cryo‑EM.	Cofactor‑mediated stabilization of active conformations.	[121],[166]
Accessory complexes (Vif–APOBEC3, NS1–host)	Structural biology, computational and cell-based approaches.	Mechanisms of immune evasion and host‑factor recruitment.	[150],[151],[167],[168]
Therapeutic implications	Drug design, VLP engineering.	Targeting capsid assembly and polymerase complexes for antiviral development.	[5],[30],[38],[41],[121],[154],[166],[171]–[173]

Abbreviations: ITC, Isothermal titration calorimetry; MD, Molecular dynamics; Cryo-EM, Cryo-electron microscopy; Vif, Viral infectivity factor; APOBEC3, Apolipoprotein B mRNA editing enzyme, catalytic polypeptide-like 3; NS1, nonstructural protein 1; VLP, Virus-like particle.

## 5. Applications

Translational impact is strongest when a biophysical observation informs a design decision and is then validated in a relevant biological or clinical setting. The examples below distinguish enabling measurements from subsequent evidence and separate licensed products from emerging platforms. The iterative workflow outlined in Figure 2 provides the framework for these applications, and Figure 3 shows links between major viral protein classes and their biophysical properties to translational applications. Table 7 compares drug discovery, conformational antigen design, virus-like-particle vaccines, and engineered capsid technologies.

**Figure 3.**
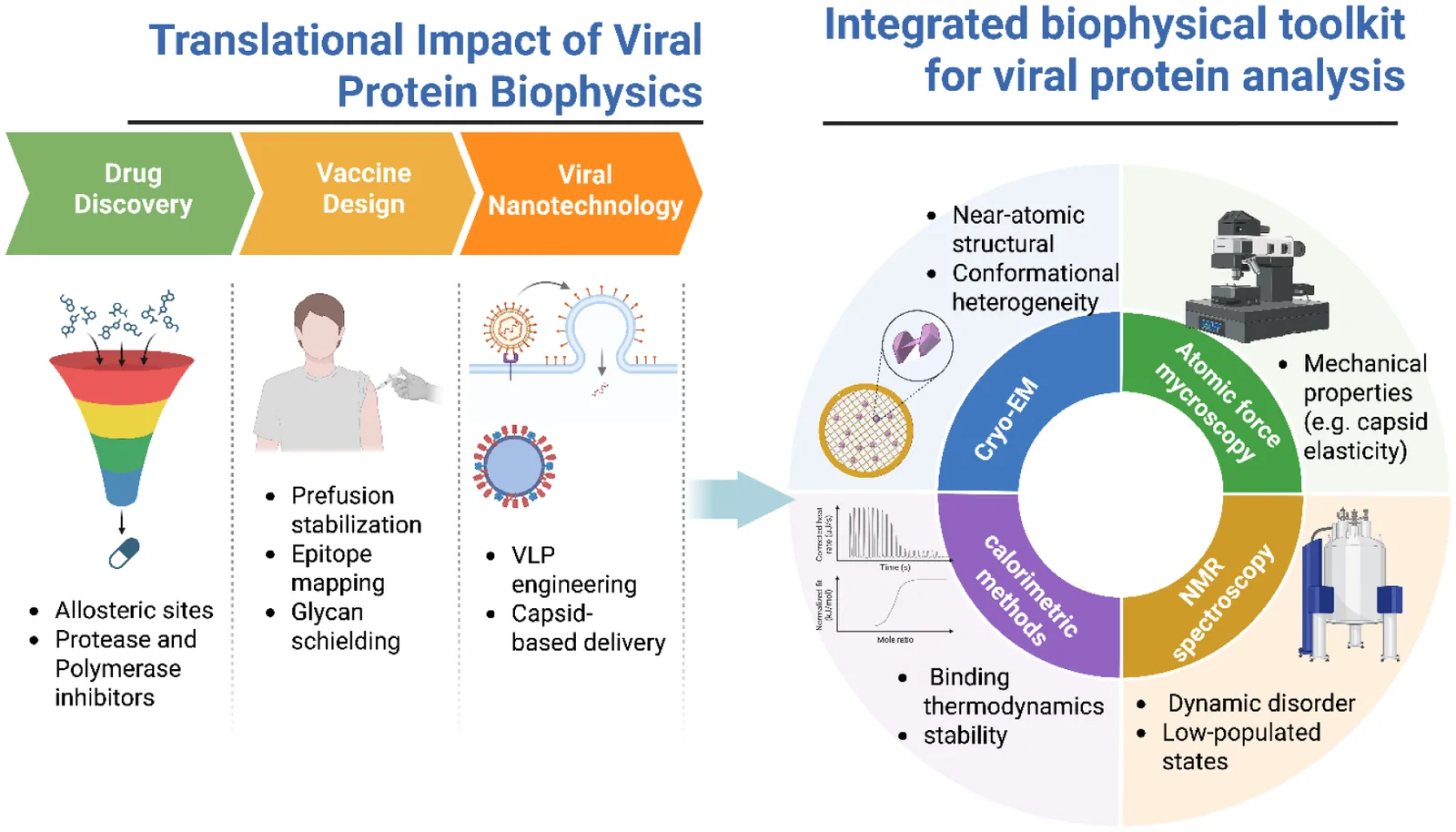
Conceptual relationship between viral protein classes, measurable biophysical properties, and translational decisions. Capsid, fusion, and regulatory proteins are linked to structural organization, conformational plasticity, mechanics, and thermodynamics, which can inform antiviral discovery, vaccine design, and viral nanotechnology. The diagram summarizes analytical routes; the strength and generality of each mechanistic claim depend on the comparative and validation criteria described in the text.

### 5.1. Drug discovery

Several antiviral classes provide direct evidence that biophysical characterization can alter medicinal chemistry. The structure and catalytic mechanism of HIV-1 protease enabled transition-state-mimetic inhibitors and established a structure-based route to the protease-inhibitor class [26],[125]. Influenza neuraminidase structures identified a conserved catalytic pocket and guided the design of zanamivir and hydrophobic analogues that ultimately led to oseltamivir [27],[28],[174]. These examples show the value of combining structural complementarity with enzymology and resistance analysis rather than optimizing affinity alone. More successes extend the same logic to dynamic and multistage targets. Nirmatrelvir was developed through structure-guided optimization of a reversible covalent inhibitor of the SARS-CoV-2 main protease; crystallography, enzyme kinetics, antiviral assays, and clinical evaluation collectively supported its progression to an approved oral therapy [29],[33]. Lenacapavir provides an orthogonal example: Structure-guided design targeted a conserved interface between HIV-1 capsid subunits, and biophysical and virological studies showed that binding perturbs nuclear import, assembly, and particle production, supporting its development as a first-in-class long-acting capsid inhibitor [30]. Because resistance can arise at both targets, structural surveillance, all-atom MD, binding thermodynamics, and phenotypic resistance assays remain necessary to distinguish reduced affinity from altered conformational populations or compensatory pathway changes. In a practical discovery workflow, cryo-EM or crystallography defines targetable states; NMR and all-atom MD can characterize or prioritize transient pockets and conformational populations; ITC, DSC, and kinetic assays quantify thermodynamic, stability, and kinetic consequences, respectively; and mutational, biochemical, and cellular assays test causality. AFM can add a mechanical readout for capsids and membranes. Broader surveys of SARS-CoV-2 targets can support prioritization, but their predictions require this multilayer validation framework [175],[176]. The key translational criterion is prospective prediction: A model should explain independent structure-activity relationships and resistance mutations, not only reproduce the complex from which it was derived.

### 5.2. Vaccine design

Structure-based vaccine design has been most successful for metastable viral fusion proteins. Stabilization of RSV F in the prefusion state preserved antigenic site Ø and elicited substantially higher neutralizing responses in animal models [31]. This work established a general antigen-engineering rule: Identify neutralization-sensitive epitopes that are lost during refolding and stabilize the conformation in which those epitopes are exposed. The prefusion-F paradigm subsequently informed the development of a vaccine antigen that demonstrated efficacy in a phase 3 trial in older adults [34]. For coronaviruses, prefusion stabilization and cryo-EM analysis of spike provided a parallel framework for antigen design. Structure-guided proline substitutions, including the HexaPro construct, reduced the tendency of spike to refold while improving expression and thermal stability [132],[177]. Although individual vaccine products differ in sequence, formulation, and delivery platform, the shared biophysical principle is the preservation of neutralization-relevant prefusion conformations. NMR, hydrogen-deuterium exchange, calorimetry, and all-atom MD can provide complementary information on conformational flexibility, solvent or glycan-mediated protection, and antigen stability that is not captured by a single density map. These examples also define important limits. Greater antigen stability is not automatically equivalent to broader protection; epitope accessibility, B-cell repertoire, antigen processing, and variant evolution must be evaluated experimentally. Structural vaccinology is therefore most predictive when high-resolution structures are combined with serology, neutralization, antigenic mapping, and *in vivo* protection rather than used as a stand-alone design endpoint.

### 5.3. Nanotechnology

VLPs provide a direct translational application of capsid assembly biophysics. Recombinant hepatitis B surface antigen self-assembles into particles that preserve repetitive antigen display [171], and HPV L1 forms highly immunogenic VLPs containing conformational epitopes [41]. Licensed HBV and HPV vaccines demonstrate that assembly geometry, particle stability, and multivalent presentation can be converted into durable vaccine platforms. Their success shows why monomeric antigen characterization is insufficient: Quaternary organization and manufacturing consistency are integral to the immunogen. Emerging dengue and SARS-CoV-2 VLP platforms extend the concept but remain at earlier stages of development and validation. Tetravalent dengue VLP design must balance expression and antigenic representation across serotypes, while coronavirus VLPs require coordinated assembly and native-like display of membrane proteins [172],[173]. Cryo-EM and antigenic mapping assess architecture and epitope exposure; light scattering, calorimetry, and AFM test assembly, thermal stability, and mechanics; and coarse-grained MD can explore how subunit affinity, membrane composition, or linker design affect morphology. VLP optimization should therefore report particle heterogeneity, antigen density, stability during storage, and preservation of neutralization-relevant epitopes, followed by serology and protection studies. Structural uniformity alone does not establish immunogenicity, and high antibody titers do not guarantee balanced or durable protection. Licensed and preclinical examples are distinguished throughout to avoid conflating platform feasibility with clinical validation.

**Table 7.** Biophysical applications in antiviral drug discovery, conformational antigen design, virus-like-particle vaccines, and engineered viral nanotechnology.

Application area	Biophysical techniques	Main contributions	Mechanistic insights	Translational implications	References
Drug discovery	ITC, DSC, AFM, all-atom MD, cryo-EM, high-throughput calorimetry, and machine learning.	Definition of druggable states; thermodynamic and kinetic profiling; mapping of resistance-sensitive interactions; mechanical readouts for capsid and membrane targets.	Structure-activity relationships, conformational mechanisms of resistance, and coupling between ligand binding and viral protein function.	Approved protease, neuraminidase, and main-protease inhibitors; development of capsid inhibitors; resistance-aware lead optimization.	[5],[26]–[30],[33],[78],[123],[125],[174],[178]–[180]
Vaccine design	Cryo-EM, NMR, all-atom MD, calorimetry, serology, and neutralization assays.	Stabilization of prefusion conformations; mapping of neutralization-sensitive epitopes; quantification of antigen dynamics and stability.	Preservation of transient prefusion epitopes and identification of stability-immunogenicity trade-offs.	Structure-guided vaccine antigens; preservation of prefusion epitopes; improved stability and immunogenicity with clinical validation.	[31],[32],[34],[132],[177],[181]–[184]
Nanotechnology	AFM, cryo-EM, all-atom MD, CGMD, calorimetry, and light scattering.	Engineering of capsids and VLPs; optimization of cargo loading and multivalent display; assessment of mechanical resilience and heterogeneity.	Relationships among subunit affinity, curvature, stiffness, cargo release, and surface presentation.	Development-stage delivery systems for drugs, nucleic acids, and antigens; explicit evaluation of manufacturability and *in vivo* performance.	[5],[15],[44],[164],[165],[185]–[187]

Abbreviations: ITC, Isothermal titration calorimetry; DSC, Differential scanning calorimetry; AFM, Atomic force microscopy; MD, Molecular dynamics; cryo-EM, Cryogenic electron microscopy; NMR, Nuclear magnetic resonance; CGMD, Coarse-grained molecular dynamics; VLPs, Virus-like particles.

## 6. Challenges and future perspectives

### 6.1. Methodological limitations

Despite major advances, each method samples only part of the relevant landscape. Cryo-EM resolution is often nonuniform: Flexible loops, glycans, membrane-proximal regions, and low-occupancy states may remain unresolved even when the global map is near-atomic. Vitrification, preferred orientation, particle classification, and conformational selection can alter or discretize continuous heterogeneity [11],[66],[188]. NMR provides residue-level dynamics but is constrained by size, spectral overlap, labeling requirements, and exchange broadening. AFM measurements depend on surface immobilization, tip geometry, loading rate, and the chosen indentation model, while ITC and DSC require relatively large amounts of homogeneous material and may not resolve binding kinetics or multiple coupled equilibria [15],[16]. Computational resolution does not remove these uncertainties. All-atom MD is limited not only by cost and sampling but also by force-field accuracy, protonation states, treatment of metal ions and glycans, and the composition of viral and host membranes. Enhanced sampling can accelerate rare transitions, but results depend on collective variables and reweighting assumptions. Coarse-grained MD reaches larger systems and longer timescales at the cost of smoother energy landscapes, reduced chemical specificity, and kinetics that are not always physically interpretable [20],[82],[85],[189]. AI-predicted structures can supply hypotheses, but confidence scores do not establish state populations, ligand ability, or function. Transient viral intermediates remain a cross-cutting challenge because they are short-lived, asynchronous, and often coupled to membranes, receptors, proteolysis, or changing pH. Time-resolved cryo-EM, NMR exchange methods, smFRET, and rapid biochemical triggering can enrich such states; yet, synchronization and ensemble averaging may still distort their apparent populations. A robust strategy is therefore to seek orthogonal validation: Models should reproduce independent structural, dynamic, mechanical, thermodynamic, and functional observables and should state the resolution and timescale over which each conclusion applies.

### 6.2. Linking molecular biophysics to microbiological variation and surveillance

Sequence surveillance and molecular epidemiology identify mutations that rise in frequency; biophysics can test which of them alter receptor usage, fusion activation, antigenic recognition, capsid stability, or drug susceptibility [104],[118],[155],[179],[190]. This link is essential for microbiological interpretation. A recurrent mutation is not necessarily adaptive, and a change in binding energy is not equivalent to increased transmission. Causality requires representative sequence sampling, phylogenetic control, structural or dynamic analysis, and phenotypic measurements in relevant cells or organisms. Multi-omics can provide the cellular context needed to interpret these molecular changes. Proteomics and transcriptomics identify host dependencies, while metabolomics and interactomics reveal state-dependent consequences [191]–[195]. Integration is challenging because measurements differ in scale, timing, and sampling. A useful model should specify how a molecular perturbation propagates to a cellular phenotype and should be tested across strains, hosts, or environments rather than being inferred from correlation alone.

### 6.3. AI and ML

AI contributes at several distinct stages. AlphaFold predicts protein structures, RoseTTAFold jointly models structures and interactions, and evolutionary scale modeling fold (ESMFold) uses protein language models for rapid structure prediction [107]–[109]. Cryo-EM Deep Reconstructing Generative Networks (CryoDRGN) uses neural networks to reconstruct continuous heterogeneity from cryo-EM particles [110]. ML classifiers can identify conformational states in simulation data, prioritize resistance-associated variants, and guide capsid-assembly analysis [23],[101]. De novo design has also produced experimentally validated SARS-CoV-2-binding miniproteins, demonstrating that AI-assisted design can generate testable antiviral molecules [111]. These tools have distinct failure modes. Structure predictors may be confident in a single fold yet omit alternative states, ligands, post-translational modifications, or membrane context. Generative and language models inherit biases from sequence and structural databases; cryo-EM latent-space methods can organize heterogeneity without establishing a kinetic pathway. AI outputs should therefore enter the same validation loop as other models: Prospective mutation, binding, dynamics, biochemical, and infection tests are required before a prediction is treated as a mechanistic explanation or clinical evidence.

### 6.4. Translational and evidentiary priorities

The next advances will depend less on adding isolated high-resolution structures than on their prospective validation. Candidate mechanisms should predict an independent mutation, state population, force response, binding energy, neutralization phenotype, or resistance profile. Models should also be tested across viral backgrounds and relevant environments before being used to infer host range, transmission, or clinical benefit. This evidentiary ladder includes molecular observation, orthogonal validation, infection phenotype, and, where appropriate, clinical or surveillance confirmation, and provides a practical standard for translation. Table 8 summarizes the key methodological, microbiological, computational, and translational challenges that limit this progression.

## 7. Conclusions

Molecular biophysics contributes to virology when it connects a measurable molecular property to a defined biological phenotype. In this review, those microbiological phenotypes include tropism, entry efficiency, environmental persistence, strain-level immune escape, and antiviral resistance. The strongest conclusions arise from orthogonal measurements and from comparisons across viral families; data-rich systems such as HIV-1 and SARS-CoV-2 are useful exemplars, but they do not by themselves establish universality. The translational record illustrates the power and the limits of the field. HIV-1 protease and capsid inhibitors, influenza neuraminidase inhibitors, SARS-CoV-2 main protease inhibitors, prefusion-stabilized antigens, and licensed HBV and HPV VLP vaccines were informed by structural or biophysical insight but required enzymology, manufacturing control, resistance analysis, immunology, and clinical testing [26]–[31],[33],[34],[41],[132],[171]. High resolution or apparent computational stability alone does not establish druggability, immunogenicity, transmission advantage, or efficacy. A predictive framework therefore requires explicit model hierarchies and uncertainty estimates. All-atom and coarse-grained MD should be used as complementary levels of resolution, AI models should be treated as hypotheses, and every technique should be evaluated against the primary observable it reports and the inference that observable supports. Linking these models to biochemical reconstitution, infection assays, sequence surveillance, and clinical data offers a realistic route from molecular mechanism to microbiological and clinical application.

**Table 8.** Key methodological, microbiological, computational, and translational challenges.

Domain	Main challenges	Priorities and opportunities
Methodological approaches	Nonuniform cryo-EM resolution, vitrification and preferred-orientation artifacts, and conformational classification bias [11],[66],[188]; NMR size, overlap, labeling, and exchange-broadening constraints [46],[47],[80]; AFM surface, tip, loading-rate, and model dependence [15]; ITC/DSC sample requirements and limited kinetic resolution [16],[17]; all-atom force-field and sampling errors; CGMD loss of chemical specificity and uncertain kinetics; difficulty capturing short-lived viral intermediates [68],[79],[189],[196].	Time-resolved and orthogonal measurements; improved cryo-EM specimen preparation and detectors; advanced NMR labeling and exchange methods; calibrated AFM and calorimetric assays; experimentally restrained all-atom and coarse-grained ensembles; prospective biochemical and cellular validation of transient states and resistance mechanisms.
Microbiological variation and multi-omics integration	Sampling and phylogenetic bias; weak linkage between sequence frequency, molecular effect, and transmission; heterogeneous omics scales and time points [155],[190]–[194].	Combine surveillance with structural, dynamic, biochemical, and infection assays; test mechanisms across strains, hosts, and environments; and move beyond purely correlational integration toward mechanistically interpretable and experimentally testable multi-omics models [191]–[195].
AI and computational modeling	Training-set bias, uncertain alternative states, force-field and sampling limitations, and insufficient reporting or prospective validation [79],[107]–[110],[189],[196].	Use AlphaFold, RoseTTAFold, ESMFold, cryoDRGN, ML state analysis, and de novo design as hypotheses within experimentally constrained workflows [23],[101],[107]–[111].
Translational applications	Rapid viral evolution, pathway redundancy, and failure to distinguish molecular plausibility from clinical actionability.	Prospective prediction of resistance, immunogenicity, or infection phenotype; explicit evidence ladders from molecular observation to clinical or surveillance confirmation [26]–[31],[33],[34],[41],[132],[171].

Abbreviations: cryo-EM, Cryogenic electron microscopy; NMR, Nuclear magnetic resonance; AFM, Atomic force microscopy; ITC, Isothermal titration calorimetry; DSC, Differential scanning calorimetry; CGMD, Coarse-grained molecular dynamics; AI, Artificial intelligence; ML, Machine learning.

## Use of AI tools declaration

The authors confirm that no artificial intelligence tools were used to generate scientific hypotheses, analyze data, or formulate conclusions. Generative artificial intelligence tools were used solely for language editing, organization of the revision, and assistance in preparing the conceptual figure. All scientific claims, references, interpretations, and final wording were independently verified by the authors, who take full responsibility for the content of the manuscript.
